# SUMOylation in Glioblastoma: A Novel Therapeutic Target

**DOI:** 10.3390/ijms20081853

**Published:** 2019-04-15

**Authors:** Brandon M. Fox, Andrew Janssen, Dagoberto Estevez-Ordonez, Florian Gessler, Nunzio Vicario, Gustavo Chagoya, Galal Elsayed, Houman Sotoudeh, William Stetler, Gregory K. Friedman, Joshua D. Bernstock

**Affiliations:** 1 Department of Neurosurgery, University of Alabama at Birmingham, 1060 Faculty Office Tower, 510 20th Street South, Birmingham, AL 35223, USA; bmfox@uab.edu (B.M.F.); janssen@uab.edu (A.J.); dagoestevez@gmail.com (D.E.-O.); gchagoya@uabmc.edu (G.C.); gelsayed@uabmc.edu (G.E.); wstetler@uabmc.edu (W.S.); GFriedman@peds.uab.edu (G.K.F.); 2 Medical Scientist Training Program, University of Alabama at Birmingham, 1825 University Boulevard, SHEL 121, Birmingham, AL 35294, USA; 3 Department of Neurosurgery, University Hospital Frankfurt, Goethe-University, Schleusenweg 2-16, 60528 Frankfurt, Germany; flo.gessler@gmail.com; 4 Department of Biomedical and Biotechnological Sciences, Physiology Section, University of Catania, Via S. Sofia n. 97, Torre Biologica, 95123 Catania, Italy; vicarionunzio@gmail.com; 5 Division of Neuroradiology, Department of Radiology, University of Alabama at Birmingham, Jefferson Tower N419—619 19th Street South, Birmingham, AL 35223, USA; HSotoudeh@uabmc.edu; 6 Division of Pediatric Hematology and Oncology, Department of Pediatrics, University of Alabama at Birmingham, Lowder 512, 1600 7th Avenue South, Birmingham, AL 35223, USA

**Keywords:** glioblastoma (GBM), SUMOylation, SUMO1-3, post-translational modifications (PTMs)

## Abstract

Protein SUMOylation is a dynamic post-translational modification which is involved in a diverse set of physiologic processes throughout the cell. Of note, SUMOylation also plays a role in the pathobiology of a myriad of cancers, one of which is glioblastoma (GBM). Accordingly, herein, we review core aspects of SUMOylation as it relates to GBM and in so doing highlight putative methods/modalities capable of therapeutically engaging the pathway for treatment of this deadly neoplasm.

## 1. Introduction

Glioblastoma (GBM), a WHO grade IV glioma, represents the most common primary brain tumor in the adult population. The current standard of care for newly diagnosed GBM includes maximal safe surgical resection with concomitant radiation followed by adjuvant chemotherapy with the alkylating drug temozolomide, which may now be combined with intermediate-frequency alternating electrical fields [1]. Unfortunately, these tumors have a dismal prognosis with a median overall survival of only 10–20 months, and individual survival rarely exceeds two years [2]. GBM is an extremely invasive and malignant tumor with a litany of characteristics that impede effective clinical treatment (e.g., GBMs are highly vascular, rapidly dividing tumors that commonly recur despite aggressive multimodal treatment regimens) [2,3]. Accordingly, new approaches to treatment are vital in an attempt to improve outcomes. An improved understanding of molecular pathways within these highly heterogeneous tumors may ultimately provide detailed insight into the driving forces behind GBM’s unusually aggressive and treatment-resistant nature. In line with such thinking, herein, we examine protein post-translational modification (PTM) with small ubiquitin-like modifier (SUMO) in the setting of GBM and discuss the therapeutic potential of targeting this pathway as a novel treatment paradigm for this devastating tumor of the central nervous system (CNS).

## 2. SUMOylation 

The regulation of cellular physiology is coordinated in part by control of protein activity, abundance, localization, and/or interactions via a variety of reversible PTMs (e.g., SUMO, phosphorylation, and ubiquitin). SUMO is a protein with ~18% homology to ubiquitin. The conjugation of SUMO has been termed SUMOylation. It is an essential PTM that is highly conserved among eukaryotic organisms [4,5]. SUMOylation occurs at a similar frequency to other major PTMs, with greater than 3600 SUMOylated human proteins identified to date [6]. Specifically, many proteins related to both the cell cycle and DNA repair pathways are known SUMOylation targets, and accordingly, this PTM plays a key role in the maintenance of genomic stability, leading to considerable interest in understanding the SUMO pathway in cancer [7,8,9]. The SUMO proteins are ~100 amino acids in length and have considerable structural overlap with ubiquitin, despite limited sequence homology [10]. In mammals, there are four known SUMO isoforms: SUMOs 1–4 [4,11,12,13,14,15,16,17]. SUMO-2 and SUMO-3 share ~95% homology and display a great deal of functional overlap [18]. However, SUMO-2 expression is considerably higher than SUMO-3, and SUMO-2 knockout mice are embryonically lethal while SUMO-3 knockout mice are phenotypically normal [19]. SUMO-1 has ~50% homology with SUMO-2/3 and is also expressed at markedly lower levels than SUMO-2 [18,19]. SUMO-1 knockout mice are viable, yet there is an adult phenotype suggesting that SUMO-2/3 can compensate for the loss of SUMO-1 in development but not in all physiological functions [20,21,22,23]. While SUMO-1 and SUMO-2/3 have both distinct as well as shared protein substrates and their conjugation dynamics vary through the cell cycle and in response to stressors [24]. SUMO-4, the least studied SUMO isoform, displays ~86% homology with SUMO-2 and likely does not mature or undergo conjugation under normal conditions [25]. Despite this, several studies have suggested that SUMO-4 is in fact conjugated under select conditions of biological stress [26,27].

As ubiquitin-like proteins, the enzymatic cascade responsible for SUMO processing and conjugation are similar to those of ubiquitination. SUMO proteins are translated in an inactive form that undergoes maturation via endopeptidase cleavage by SUMO-specific proteases, also known as sentrin-specific proteases (SENPs) prior to entering the conjugation cascade [28,29]. The proteins that participate in SUMO conjugation are classified as E1 activating enzymes, an E2 conjugation protein, and E3 target protein ligases. Conjugation begins with activation of mature SUMO by the E1 complex, a heterodimer of SUMO-activating enzyme 1 and 2 (SAE1, SAE2) [24,30]. Activation proceeds via a two-step ATP-dependent reaction that concludes with a high-energy thioester bond between the C-terminus of SUMO and the E1 complex [31]. Following activation, the SUMO-specific conjugating enzyme Ubc9, the sole E2, binds to E1-SUMO intermediate via an interaction with SAE2 [32,33]. Next, SUMO is transferred from the E1 complex to the cysteine residue of the Ubc9 active site, and subsequently, Ubc9 catalyzes the formation of an isopeptide bond with a lysine residue of the substrate protein, thus completing conjugation [34,35,36]. Notably, Ubc9 facilitates substrate targeting through recognition of a consensus motif that includes the target lysine [37,38]. In a substrate-specific manner, the final step of conjugation may also require the assistance of an E3 protein ligase to facilitate interaction of Ubc9 with substrate. Human SUMO E3 ligases include the protein inhibitor of activated STAT (PIAS) family and RanBP3, both of which function with a wide variety of substrates, as well as a variety of other E3 ligases that function with a narrower collection of substrate proteins [39,40,41,42]. The final component of the SUMOylation pathway is deconjugation or deSUMOylation. Like maturation, deconjugation is catalyzed by SENPs, albeit via isopeptidase activity instead of endopeptidase activity [28,29]. Importantly, the balance between SUMO conjugation and deconjugation determines protein SUMOylation status, and this process is in part controlled by the regulation of the activity and localization of SENPs [43]. In humans, there are six SENPs, SENP1-3 and SENP5-7 [44,45]. Additionally, two other SUMO-specific proteases, USPL1 and DeSI1, have been identified, though the scale of their role in broader de-SUMOylation appears to be limited [46,47]. Individual SENPs exhibit unique SUMO protein specificity, localization, and affinity for endopeptidase versus isopeptidase activity.

## 3. SUMOylation and Cancer

In cancer, dysregulation of PTMs plays an important role in the pathologic cellular processes that underlie malignant transformation and progression. SUMOylation was first associated with cancer not long after its discovery, with the identification of promyelocytic leukemia protein (PML) as one of the first known SUMOylation substrates [48]. In acute promyelocytic leukemia (APL), a chromosomal translocation involving chromosomes 15 and 17 leads to the creation of the PML-retinoic acid receptor-α (PML-RARα) fusion oncoprotein [49,50]. Pathologic APL blasts are deficient in structures known as PML nuclear bodies, which require SUMOylation of PML in order to form [51]. Curative treatment of APL is accomplished with arsenic compounds that cause poly-SUMOylation of PML-RARα, which facilitates degradation of the oncoprotein and restoration of PML bodies [52,53,54]. These discoveries in APL provided an important proof-of-concept for the role of SUMOylation in cancer and opened the door for a broad investigation into the PTM’s role in many other cancers.

Genomic stability is maintained through the coordinated action of both the DNA damage response (DDR) and cell cycle regulation. These vital cellular processes prevent the accumulation and passage of somatic mutations and safeguard against malignant transformation. DNA replication and repair proteins are critical functional components of the DDR, and many of these proteins are known to be SUMOylation substrates [55]. Additionally, the SUMOylation of DDR proteins increases in response to DNA damage, and further, functionally necessary SUMOylation events have been identified in the setting of DNA repair [56,57,58,59,60]. Normal cell cycle regulation also requires the SUMOylation pathway, with many cyclins and cyclin-dependent kinases (CDKs) having been identified as SUMOylation substrates [61,62,63]. In the setting of cancer, dysregulated SUMOylation of CDKs prevents normal ubiquitin-mediated degradation, thus allowing CDK persistence and pathological cell cycle progression [64]. Finally, many oncoproteins and tumor suppressors promote carcinogenesis through the enhancement, absence, or dysfunction of their actions in the cell cycle or the DDR, and SUMOylation has been implicated in the function of a number of these cancer-associated proteins including p53, RB, BRCA1, MYC, MDM2, and cyclin D1 [59,65,66,67,68,69]. Taken together, prior studies have established a critical role of the SUMOylation pathway in the maintenance of genomic stability and have highlighted that dysregulated SUMOylation places genomic integrity at risk.

A growing body of evidence supports the concept that SUMOylation is broadly enhanced in many types of cancer. Upregulation of SUMO proteins, SUMOylation machinery (e.g., Ubc9), and SUMO ligases have been identified in a variety of malignancies including brain, lung, GI, liver, pancreas, breast, prostate and skin as well as lymphoma and multiple myeloma [64,70,71,72,73,74,75,76,77,78,79]. While upregulation of the SUMOylation pathway appears the predominant perturbation in the setting of cancer, it is prudent to note that downregulation has been reported [76]. Further, upregulation of SUMO-specific proteases in various malignancies suggests that dysregulation of de-SUMOylation is also present in numerous cancers [7,80]. Considering the robust alteration in the SUMOylation pathway across diverse cancers, it is perhaps not surprising that many of the defining characteristics of malignancy have been linked to SUMOylation, including apoptotic resistance, replicative immortality, angiogenesis, invasion, and metastasis [69,81,82,83,84]. Considering these multi-level biological connections between SUMOylation and cancer that extend across diverse malignancies, it is plausible that dysregulated SUMOylation is fundamental in malignancy, and it has been proposed that SUMOylation-mediated stress resistance may thus represent a unifying characteristic [80,85]. 

## 4. SUMOylation in Glioblastoma

As noted above, GBM continues to portend a dismal prognosis despite an improved understanding of the underlying biology of GBM at the molecular level (e.g., IDH1 mutational status and *MGMT* methylation status). Research advances in GBM have revealed that a multitude of cellular signaling pathways are dramatically altered by the disease, and due to this complexity, targeting cellular signaling pathways with wide-ranging biological actions, such as PTMs, may be necessary to achieve more robust treatment responses [86]. As SUMOylation targets thousands of proteins and participates in many critical cellular processes, especially in conditions of stress, this PTM may represent such a target.

**Figure 1:**
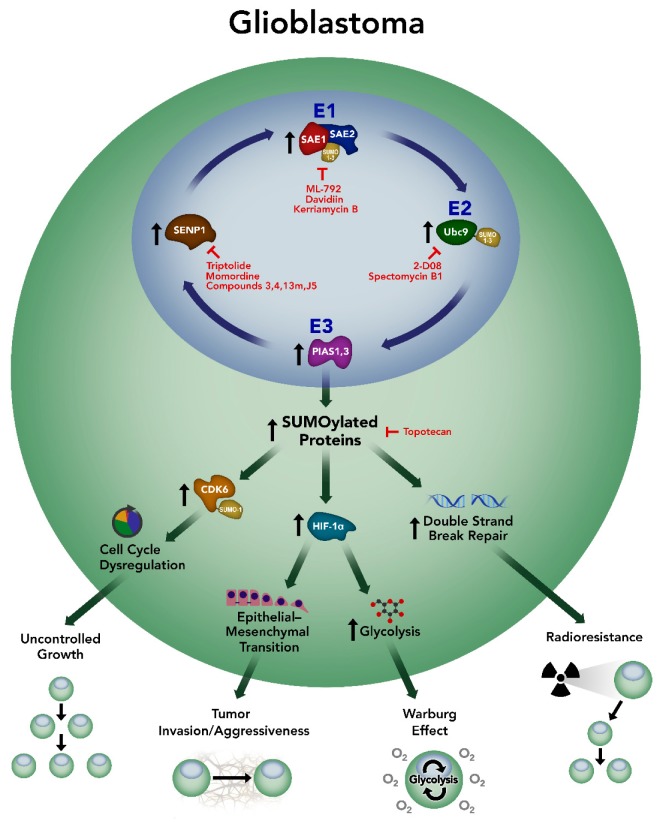
Enhancement of SUMOylation in glioblastoma contributes to alterations in multiple cellular processes leading to an increased malignant phenotype. Glioblastoma displays an increase in E1 (SAE1), E2 (Ubc9), and E3 (PIAS1 and PIAS3) enzymes leading to global enhancement of SUMOylation and a resultant increase in the SUMOylation level of target proteins. Further, increased expression of SUMO-specific protease (SENP1) has been reported in glioblastoma. Presently, studies have implicated CDK6 and HIF-1α as important loci downstream of these effects that together contribute to cell cycle dysregulation, epithelial-mesenchymal transition, and heightened glycolytic metabolism. DNA double-strand break repair has additionally been associated with perturbations in SUMOylation. Together, these altered cellular processes give rise to an enhanced malignant phenotype in glioblastoma including uncontrolled growth, increased invasion and aggressiveness, a malignant bioenergetics profile with Warburg effect, and resistance to ionizing radiation. Targeting SUMOylation may represent a therapeutic approach to reverse the pathologic consequences of enhanced SUMOylation in glioblastoma. Available therapeutic agents known to inhibit SUMOylation are identified along with their targets in the SUMOylation pathway.

Astrocytic malignancies, including GBM, were one of the first cancers in which global upregulation of SUMOylation was identified [70]. Yang et al. demonstrated a nearly 30-fold increase in the level of SUMO-1 and SUMO-2/3 conjugated proteins in patient-derived GBM samples. SUMOylation was also significantly upregulated in grade II and III astrocytomas but was highest in GBM [70]. Importantly, upregulation of SUMOylation in GBM has since been replicated in both patient samples and multiple human GBM cell lines [64,87]. Components of the SUMOylation pathway are also upregulated in both GBM samples and cell lines, including E1 (SAE1), E2 (Ubc9), and E3 (PIAS1 and 3) components as well as a SUMO-specific protease (SENP1) (Figure 1) [64,70,88,89,90]. Taken together, these studies provide strong evidence that SUMOylation is enhanced/aberrant in GBM.

To initially characterize the role of upregulated SUMOylation in GBM, Yang et al. utilized gene silencing of SUMO-1-3 and demonstrated disruption of DNA synthesis and cell growth as well as decreased clonogenic survival [70]. This study additionally found that silencing SUMO-1-3 in GBM interfered with the DDR, specifically double-strand break repair, which is consistent with prior studies of SUMOylation in DNA repair mechanisms [70]. This deficiency suggests that enhancement of SUMOylation acts to protect GBM cells from DNA damage, thus promoting survival and potentially contributing to radiation resistance. To this effect, Soars et al. examined the impact of the SUMOylation pathway on GBM sensitivity to radiation. They demonstrated upregulation of the SUMO-specific E3 ligase PIAS1 in a human GBM cell line and showed that radiation causes PIAS1 to interact with stress-inducible phosphoprotein 1 (STI1) leading to the protein’s nuclear accumulation and ultimately resistance to radiation-induced cell death [88]. Together these studies implicate upregulation of the SUMOylation pathway in pro-survival processes and suggest that the pathway may contribute to radioresistance in GBM.

The SUMOylation pathway is known to contribute to cell cycle regulation via the SUMOylation of cyclins and CDKs [61,62,63]. Bellail et al. demonstrated that activation SUMOylation in GBM leads to SUMO-1 adduction to CDK6 [64]. Under normal conditions, CDK6 ubiquitination and proteasomal degradation lead to controlled cell cycle arrest at the G1 checkpoint [91]. In GBM, SUMO-1 modified CDK6 does not undergo ubiquitination, and as a result, the protein is stabilized and drives the cell cycle through the G1-S transition. This process releases the brake on the cell cycle in GBM, as knockdown of either SUMO-1 or CDK6 in GBM cells or xenografts leads to growth inhibition. Further, patient samples were found to have elevations in both SUMO-1 and CDK6 [64]. In support of these findings, we confirmed the SUMO-1-CDK6 interaction in GBM cells and demonstrated that small molecule-mediated inhibition of SUMOylation can decrease in CDK6 levels in multiple human GBM cell lines [87]. Of note, the decrease in CDK6 in response to inhibition of SUMOylation was mediated by proteasomal degradation, consistent with the mechanism that SUMO-1 conjugation to CDK6 prevents its ubiquitin-mediated degradation [87]. Together these studies demonstrate that enhanced SUMOylation in GBM acts directly on the cell cycle, ultimately leading to uncontrolled tumor growth.

As a PTM with thousands of substrates, SUMOylation is known to affect a multitude of cellular processes. In an effort to examine how SUMOylation modifies the functional landscape of GBM we recently utilized liquid chromatography (LC)/mass spectrometry (MS)/MS analysis to examine changes in SUMO-1 conjugated proteins in response to inhibition of SUMOylation [87]. This analysis revealed multiple pathways that are affected by SUMOylation in GBM including DNA double-strand break repair and apoptosis signaling, consistent with prior studies [70,87,88]. However, the analysis also revealed that in GBM SUMOylation alters cellular metabolism through enhancement of glycolysis and the pentose-phosphate pathway, consistent with prior studies of SUMOylation-mediated effects on metabolism as well as the bioenergetics phenotype of GBM [92,93,94]. These alterations were likely related to the effect of SUMOylation on hypoxia-inducible factor-1 (HIF-1α) that was demonstrated in the study. Of note, the SUMOylation-mediated stabilization of HIF-1α identified in the study may also have implications for GBM progression via its effect on epithelial-mesenchymal transitions (EMT). HIF-1α promotes EMT-associated protein expression, and in GBM, EMT is associated with progression and acquisition of a highly invasive phenotype [95,96,97]. Finally, we also demonstrated that in human GBM tissue samples there is wide variation in global SUMOylation levels [87]. Differences in SUMOylation and resultant downstream effects, as discussed above, may in part explain phenotypic differences between patients, such as the presence of an aggressive mesenchyme phenotype. Thus, assessment of SUMOylation status may ultimately aid in prognostication and/or in future personalized GBM therapies.

## 5. Targeting SUMOylation in Glioblastoma 

Because of the important role SUMOylation plays in GBM, the pathway has significant potential as a novel therapeutic target to treat this intractable malignant neoplasm. Critically, several new natural and synthetic small molecule inhibitors of SENPs, SAE1, and Ubc9 have been described in the literature [29,98,99,100,101,102]. Although many are new and/or still in early experimental phases, they have significant potential, and several studies have explored the effect of targeting key proteins in SUMOylation in astrocytoma and GBM.

Several Food and Drug Administration (FDA) approved drugs have been assessed as SUMOylation pathway targets. Topotecan is a semisynthetic water-soluble drug from the camptothecin family with current approval for the treatment of several cancers (e.g., small cell lung cancer, cervical, ovarian) [103,104]; it is primarily a DNA topoisomerase I inhibitor. However, it also modulates the SUMOylation status of its primary target and has been shown to inhibit HIF-1α [105,106,107]. In GBM, topotecan was recently found to inhibit global SUMOylation and as a result, reduce both levels of CDK6 and HIF-1α thereby inducing pronounced changes in cell cycle progression and cellular metabolism [87]. 

Several other drugs have also been identified that interact with the SUMOylation pathway [29,85,98,108,109,110]. Although many of these have no data in GBM models as of yet, they all harbor potential as therapeutic targets in GBM. Spectomycin B1 has also been identified as a SUMOylation inhibitor by directly binding Ubc9 [108]. SAE1/2 inhibitors, such as ML-792 have been shown to potently inhibit SUMOylation with a promising application in treating MYC-amplified malignancies [111,112]. Other SAE1 targets that result in SUMOylation inhibition include ginkgolic acid [113], kerramycin B [114], davidiin [115], and tannic acid [116]. 

Of note, the rational design of SENP inhibitors is also technically possible [29]. Xia et al. showed that downregulation of SENP1 in astrocytoma and GBM led to inhibition of the phosphorylation of IκBα and Akt, and also the expression of its downstream regulation factors Bcl-xL and cyclinD1 [90]. Drugs or biologic agents designed to target inhibition of SENP1 could thus promote and induce apoptosis in GBM by regulating NF-κB/Akt pathways. Other SENP1 inhibitors that have been identified, albeit without data in GBM, include triptolide [99], momordine [117], compound J5, compound 4, compound 3, and compound 13m [98]. 

Beyond small molecules, genetic manipulation of the pathway is also feasible. MicroRNAs (e.g., miRNA-182 and 183) have been shown to suppress the SUMOylation pathway and as such, harbor pharmaceutical potential in malignancies defined by upregulation [110]. Mechanistically, activation of SUMO requires ATP condensation of its C-terminal tail [118]. This important step is catalyzed by SAE which recognizes the C-terminus [118]. Zhao et al. described using phage display to show that a broad profile of SUMO C-terminal sequences could be activated by SAE [119]. They subsequently developed SUMO-mimicking peptides that were conjugated to SAE and Ubc9 and blocked full-length SUMO from entering the cascade [119]. Such strategies represent further examples of how SUMOylation may be targeted for therapeutic benefit in GBM.

## 6. Conclusions

GBM is the most common/aggressive primary CNS tumor within the adult population, and there have been limited advances to improve outcomes for this deadly malignancy [2]. Accordingly, there is a great need to develop novel therapeutic approaches to reduce overall morbidity and mortality for GBM patients. SUMOylation, which plays an important role in a myriad of normal cellular processes and is involved in maintaining cellular homeostasis, has also been shown to play a vital role in pathological processes (e.g., allowing CNS tumors to resist/withstand stressful conditions for cell survival and continued growth) [70,87]. Accordingly, inhibition of SUMOylation in GBM provides opportunities for the development of novel therapeutics and therefore warrants continued investigation.

## References

[1] Stupp R., Hegi M.E., Mason W.P., van den Bent M.J., Taphoorn M.J., Janzer R.C., Ludwin S.K., Allgeier A., Fisher B., Belanger K. (2009). Effects of radiotherapy with concomitant and adjuvant temozolomide versus radiotherapy alone on survival in glioblastoma in a randomised phase III study: 5-year analysis of the EORTC-NCIC trial. Lancet Oncol..

[2] Polivka J., Polivka J., Holubec L., Kubikova T., Priban V., Hes O., Pivovarcikova K., Treskova I. (2017). Advances in Experimental targeted therapy and immunotherapy for patients with glioblastoma multiforme. Anticancer Res..

[3] Touat M., Idbaih A., Sanson M., Ligon K.L. (2017). Glioblastoma targeted therapy: Updated approaches from recent biological insights. Ann. Oncol..

[4] Mahajan R., Delphin C., Guan T., Gerace L., Melchior F. (1997). A small ubiquitin-related polypeptide involved in targeting RanGAP1 to nuclear pore complex protein RanBP2. Cell.

[5] Flotho A., Melchior F. (2013). Sumoylation: A regulatory protein modification in health and disease. Annu. Rev. Biochem..

[6] Hendriks I.A., Vertegaal A.C. (2016). A comprehensive compilation of SUMO proteomics. Nat. Rev. Mol. Cell Biol..

[7] Eifler K., Vertegaal A.C.O. (2015). SUMOylation-mediated regulation of cell cycle progression and cancer. Trends Biochem. Sci..

[8] Bergink S., Jentsch S. (2009). Principles of ubiquitin and SUMO modifications in DNA repair. Nature.

[9] Sarangi P., Zhao X. (2015). SUMO-mediated regulation of DNA damage repair and responses. Trends Biochem. Sci..

[10] Bayer P., Arndt A., Metzger S., Mahajan R., Melchior F., Jaenicke R., Becker J. (1998). Structure determination of the small ubiquitin-related modifier SUMO-1. J. Mol. Biol..

[11] Shen Z., Pardington-Purtymun P.E., Comeaux J.C., Moyzis R.K., Chen D.J. (1996). UBL1, a human ubiquitin-like protein associating with human RAD51/RAD52 proteins. Genomics.

[12] Boddy M.N., Howe K., Etkin L.D., Solomon E., Freemont P.S. (1996). PIC 1, a novel ubiquitin-like protein which interacts with the PML component of a multiprotein complex that is disrupted in acute promyelocytic leukaemia. Oncogene.

[13] Matunis M.J., Coutavas E., Blobel G. (1996). A novel ubiquitin-like modification modulates the partitioning of the Ran-GTPase-activating protein RanGAP1 between the cytosol and the nuclear pore complex. J. Cell Biol..

[14] Lapenta V., Chiurazzi P., van der Spek P., Pizzuti A., Hanaoka F., Brahe C. (1997). SMT3A, a human homologue of the S. cerevisiae SMT3 gene, maps to chromosome 21qter and defines a novel gene family. Genomics.

[15] Kamitani T., Nguyen H.P., Kito K., Fukuda-Kamitani T., Yeh E.T. (1998). Covalent modification of PML by the sentrin family of ubiquitin-like proteins. J. Biol. Chem..

[16] Kamitani T., Kito K., Nguyen H.P., Fukuda-Kamitani T., Yeh E.T. (1998). Characterization of a second member of the sentrin family of ubiquitin-like proteins. J. Biol. Chem..

[17] Bohren K.M., Nadkarni V., Song J.H., Gabbay K.H., Owerbach D. (2004). A M55V polymorphism in a novel SUMO gene (SUMO-4) differentially activates heat shock transcription factors and is associated with susceptibility to type I diabetes mellitus. J. Biol. Chem..

[18] Johnson E.S. (2004). Protein modification by SUMO. Annu. Rev. Biochem..

[19] Wang L., Wansleeben C., Zhao S., Miao P., Paschen W., Yang W. (2014). SUMO2 is essential while SUMO3 is dispensable for mouse embryonic development. EMBO Rep..

[20] Evdokimov E., Sharma P., Lockett S.J., Lualdi M., Kuehn M.R. (2008). Loss of SUMO1 in mice affects RanGAP1 localization and formation of PML nuclear bodies, but is not lethal as it can be compensated by SUMO2 or SUMO3. J. Cell Sci..

[21] Zhang F.P., Mikkonen L., Toppari J., Palvimo J.J., Thesleff I., Janne O.A. (2008). Sumo-1 function is dispensable in normal mouse development. Mol. Cell. Biol..

[22] Mikkonen L., Hirvonen J., Janne O.A. (2013). SUMO-1 regulates body weight and adipogenesis via PPARgamma in male and female mice. Endocrinology.

[23] Venteclef N., Jakobsson T., Ehrlund A., Damdimopoulos A., Mikkonen L., Ellis E., Nilsson L.M., Parini P., Janne O.A., Gustafsson J.A. (2010). GPS2-dependent corepressor/SUMO pathways govern anti-inflammatory actions of LRH-1 and LXRbeta in the hepatic acute phase response. Genes Dev..

[24] Bernstock J.D., Yang W., Ye D.G., Shen Y., Pluchino S., Lee Y.J., Hallenbeck J.M., Paschen W. (2018). SUMOylation in brain ischemia: Patterns, targets, and translational implications. J. Cereb. Blood Flow Metab..

[25] Owerbach D., McKay E.M., Yeh E.T., Gabbay K.H., Bohren K.M. (2005). A proline-90 residue unique to SUMO-4 prevents maturation and sumoylation. Biochem. Biophys. Res. Commun..

[26] Wei W., Yang P., Pang J., Zhang S., Wang Y., Wang M.H., Dong Z., She J.X., Wang C.Y. (2008). A stress-dependent SUMO4 sumoylation of its substrate proteins. Biochem. Biophys. Res. Commun..

[27] Baczyk D., Audette M.C., Drewlo S., Levytska K., Kingdom J.C. (2017). SUMO-4: A novel functional candidate in the human placental protein SUMOylation machinery. PLoS ONE.

[28] Hickey C.M., Wilson N.R., Hochstrasser M. (2012). Function and regulation of SUMO proteases. Nat. Rev. Mol. Cell Biol..

[29] Bernstock J.D., Ye D., Smith J.A., Lee Y.J., Gessler F.A., Yasgar A., Kouznetsova J., Jadhav A., Wang Z., Pluchino S. (2018). Quantitative high-throughput screening identifies cytoprotective molecules that enhance SUMO conjugation via the inhibition of SUMO-specific protease (SENP)2. FASEB J..

[30] Gong L., Li B., Millas S., Yeh E.T. (1999). Molecular cloning and characterization of human AOS1 and UBA2, components of the sentrin-activating enzyme complex. FEBS Lett..

[31] Olsen S.K., Capili A.D., Lu X., Tan D.S., Lima C.D. (2010). Active site remodelling accompanies thioester bond formation in the SUMO E1. Nature.

[32] Gong L., Kamitani T., Fujise K., Caskey L.S., Yeh E.T. (1997). Preferential interaction of sentrin with a ubiquitin-conjugating enzyme, Ubc9. J. Biol. Chem..

[33] Bouillot-Eimer S., Loiseau H., Vital A. (2005). Subcutaneous tumoral seeding from a glioblastoma following stereotactic biopsy: Case report and review of the literature. Clin. Neuropathol..

[34] Desterro J.M., Thomson J., Hay R.T. (1997). Ubch9 conjugates SUMO but not ubiquitin. FEBS Lett..

[35] Schwarz S.E., Matuschewski K., Liakopoulos D., Scheffner M., Jentsch S. (1998). The ubiquitin-like proteins SMT3 and SUMO-1 are conjugated by the UBC9 E2 enzyme. Proc. Natl. Acad. Sci. USA.

[36] Lee G.W., Melchior F., Matunis M.J., Mahajan R., Tian Q., Anderson P. (1998). Modification of Ran GTPase-activating protein by the small ubiquitin-related modifier SUMO-1 requires Ubc9, an E2-type ubiquitin-conjugating enzyme homologue. J. Biol. Chem..

[37] Rodriguez M.S., Dargemont C., Hay R.T. (2001). SUMO-1 conjugation in vivo requires both a consensus modification motif and nuclear targeting. J. Biol. Chem..

[38] Sampson D.A., Wang M., Matunis M.J. (2001). The small ubiquitin-like modifier-1 (SUMO-1) consensus sequence mediates Ubc9 binding and is essential for SUMO-1 modification. J. Biol. Chem..

[39] Johnson E.S., Gupta A.A. (2001). An E3-like factor that promotes SUMO conjugation to the yeast septins. Cell.

[40] Rytinki M.M., Kaikkonen S., Pehkonen P., Jaaskelainen T., Palvimo J.J. (2009). PIAS proteins: Pleiotropic interactors associated with SUMO. Cell. Mol. Life Sci..

[41] Pichler A., Gast A., Seeler J.S., Dejean A., Melchior F. (2002). The nucleoporin RanBP2 has SUMO1 E3 ligase activity. Cell.

[42] Henley J.M., Craig T.J., Wilkinson K.A. (2014). Neuronal SUMOylation: Mechanisms, physiology, and roles in neuronal dysfunction. Physiol. Rev..

[43] Yeh E.T. (2009). SUMOylation and De-SUMOylation: Wrestling with life’s processes. J. Biol. Chem..

[44] Gong L., Millas S., Maul G.G., Yeh E.T. (2000). Differential regulation of sentrinized proteins by a novel sentrin-specific protease. J. Biol. Chem..

[45] Yeh E.T., Gong L., Kamitani T. (2000). Ubiquitin-like proteins: New wines in new bottles. Gene.

[46] Schulz S., Chachami G., Kozaczkiewicz L., Winter U., Stankovic-Valentin N., Haas P., Hofmann K., Urlaub H., Ovaa H., Wittbrodt J. (2012). Ubiquitin-specific protease-like 1 (USPL1) is a SUMO isopeptidase with essential, non-catalytic functions. EMBO Rep..

[47] Shin E.J., Shin H.M., Nam E., Kim W.S., Kim J.H., Oh B.H., Yun Y. (2012). DeSUMOylating isopeptidase: A second class of SUMO protease. EMBO Rep..

[48] Sternsdorf T., Jensen K., Will H. (1997). Evidence for covalent modification of the nuclear dot-associated proteins PML and Sp100 by PIC1/SUMO-1. J. Cell Biol..

[49] Rowley J.D., Golomb H.M., Dougherty C. (1977). 15/17 translocation, a consistent chromosomal change in acute promyelocytic leukaemia. Lancet.

[50] De The H., Chomienne C., Lanotte M., Degos L., Dejean A. (1990). The t(15;17) translocation of acute promyelocytic leukaemia fuses the retinoic acid receptor alpha gene to a novel transcribed locus. Nature.

[51] Zhong S., Muller S., Ronchetti S., Freemont P.S., Dejean A., Pandolfi P.P. (2000). Role of SUMO-1-modified PML in nuclear body formation. Blood.

[52] Muller S., Matunis M.J., Dejean A. (1998). Conjugation with the ubiquitin-related modifier SUMO-1 regulates the partitioning of PML within the nucleus. EMBO J..

[53] Tatham M.H., Geoffroy M.C., Shen L., Plechanovova A., Hattersley N., Jaffray E.G., Palvimo J.J., Hay R.T. (2008). RNF4 is a poly-SUMO-specific E3 ubiquitin ligase required for arsenic-induced PML degradation. Nat. Cell Biol..

[54] Lallemand-Breitenbach V., Jeanne M., Benhenda S., Nasr R., Lei M., Peres L., Zhou J., Zhu J., Raught B., de The H. (2008). Arsenic degrades PML or PML-RARalpha through a SUMO-triggered RNF4/ubiquitin-mediated pathway. Nat. Cell Biol..

[55] Altmannova V., Kolesar P., Krejci L. (2012). SUMO wrestles with recombination. Biomolecules.

[56] Cremona C.A., Sarangi P., Yang Y., Hang L.E., Rahman S., Zhao X. (2012). Extensive DNA damage-induced sumoylation contributes to replication and repair and acts in addition to the mec1 checkpoint. Mol. Cell.

[57] Silver H.R., Nissley J.A., Reed S.H., Hou Y.M., Johnson E.S. (2011). A role for SUMO in nucleotide excision repair. DNA Repair.

[58] Maeda D., Seki M., Onoda F., Branzei D., Kawabe Y., Enomoto T. (2004). Ubc9 is required for damage-tolerance and damage-induced interchromosomal homologous recombination in S. cerevisiae. DNA Repair.

[59] Morris J.R., Boutell C., Keppler M., Densham R., Weekes D., Alamshah A., Butler L., Galanty Y., Pangon L., Kiuchi T. (2009). The SUMO modification pathway is involved in the BRCA1 response to genotoxic stress. Nature.

[60] Luo K., Zhang H., Wang L., Yuan J., Lou Z. (2012). Sumoylation of MDC1 is important for proper DNA damage response. EMBO J..

[61] Bonne-Andrea C., Kahli M., Mechali F., Lemaitre J.M., Bossis G., Coux O. (2013). SUMO2/3 modification of cyclin E contributes to the control of replication origin firing. Nat. Commun..

[62] Schimmel J., Eifler K., Sigurethsson J.O., Cuijpers S.A., Hendriks I.A., Verlaan-de Vries M., Kelstrup C.D., Francavilla C., Medema R.H., Olsen J.V. (2014). Uncovering SUMOylation dynamics during cell-cycle progression reveals FoxM1 as a key mitotic SUMO target protein. Mol. Cell.

[63] Hendriks I.A., D’Souza R.C., Yang B., Verlaan-de Vries M., Mann M., Vertegaal A.C. (2014). Uncovering global SUMOylation signaling networks in a site-specific manner. Nat. Struct. Mol. Biol..

[64] Bellail A.C., Olson J.J., Hao C. (2014). SUMO1 modification stabilizes CDK6 protein and drives the cell cycle and glioblastoma progression. Nature Commun..

[65] Carter S., Bischof O., Dejean A., Vousden K.H. (2007). C-terminal modifications regulate MDM2 dissociation and nuclear export of p53. Nat. Cell Biol..

[66] Ledl A., Schmidt D., Muller S. (2005). Viral oncoproteins E1A and E7 and cellular LxCxE proteins repress SUMO modification of the retinoblastoma tumor suppressor. Oncogene.

[67] Gonzalez-Prieto R., Cuijpers S.A., Kumar R., Hendriks I.A., Vertegaal A.C. (2015). c-Myc is targeted to the proteasome for degradation in a SUMOylation-dependent manner, regulated by PIAS1, SENP7 and RNF4. Cell Cycle.

[68] Ding B., Sun Y., Huang J. (2012). Overexpression of SKI oncoprotein leads to p53 degradation through regulation of MDM2 protein sumoylation. J. Biol. Chem..

[69] Kim J.H., Choi H.J., Kim B., Kim M.H., Lee J.M., Kim I.S., Lee M.H., Choi S.J., Kim K.I., Kim S.I. (2006). Roles of sumoylation of a reptin chromatin-remodelling complex in cancer metastasis. Nature Cell Biol..

[70] Yang W., Wang L., Roehn G., Pearlstein R.D., Ali-Osman F., Pan H., Goldbrunner R., Krantz M., Harms C., Paschen W. (2013). Small ubiquitin-like modifier 1-3 conjugation [corrected] is activated in human astrocytic brain tumors and is required for glioblastoma cell survival. Cancer Sci..

[71] Li H., Niu H., Peng Y., Wang J., He P. (2013). Ubc9 promotes invasion and metastasis of lung cancer cells. Oncol. Rep..

[72] Shao D.F., Wang X.H., Li Z.Y., Xing X.F., Cheng X.J., Guo T., Du H., Hu Y., Dong B., Ding N. (2015). High-level SAE2 promotes malignant phenotype and predicts outcome in gastric cancer. Am. J. Cancer Res..

[73] Zhang H., Kuai X., Ji Z., Li Z., Shi R. (2013). Over-expression of small ubiquitin-related modifier-1 and sumoylated p53 in colon cancer. Cell Biochem. Biophys..

[74] Guo W.H., Yuan L.H., Xiao Z.H., Liu D., Zhang J.X. (2011). Overexpression of SUMO-1 in hepatocellular carcinoma: A latent target for diagnosis and therapy of hepatoma. J. Cancer Res. Clin. Oncol..

[75] Chien W., Lee K.L., Ding L.W., Wuensche P., Kato H., Doan N.B., Poellinger L., Said J.W., Koeffler H.P. (2013). PIAS4 is an activator of hypoxia signalling via VHL suppression during growth of pancreatic cancer cells. Br. J. Cancer.

[76] Moschos S.J., Jukic D.M., Athanassiou C., Bhargava R., Dacic S., Wang X., Kuan S.F., Fayewicz S.L., Galambos C., Acquafondata M. (2010). Expression analysis of Ubc9, the single small ubiquitin-like modifier (SUMO) E2 conjugating enzyme, in normal and malignant tissues. Hum. Pathol..

[77] Oliveira Alves M.G., da Mota Delgado A., Balducci I., Carvalho Y.R., Cavalcante A.S., Almeida J.D. (2014). Study of MDM2 and SUMO-1 expression in actinic cheilitis and lip cancer. Arch. Dermatol. Res..

[78] Hoellein A., Fallahi M., Schoeffmann S., Steidle S., Schaub F.X., Rudelius M., Laitinen I., Nilsson L., Goga A., Peschel C. (2014). Myc-induced SUMOylation is a therapeutic vulnerability for B-cell lymphoma. Blood.

[79] Driscoll J.J., Pelluru D., Lefkimmiatis K., Fulciniti M., Prabhala R.H., Greipp P.R., Barlogie B., Tai Y.T., Anderson K.C., Shaughnessy J.D. (2010). The sumoylation pathway is dysregulated in multiple myeloma and is associated with adverse patient outcome. Blood.

[80] Seeler J.S., Dejean A. (2017). SUMO and the robustness of cancer. Nat. Rev. Cancer.

[81] Renner F., Moreno R., Schmitz M.L. (2010). SUMOylation-dependent localization of IKKepsilon in PML nuclear bodies is essential for protection against DNA-damage-triggered cell death. Mol. Cell.

[82] Potts P.R., Yu H. (2007). The SMC5/6 complex maintains telomere length in ALT cancer cells through SUMOylation of telomere-binding proteins. Nat. Struct. Mol. Biol..

[83] Li J., Xu Y., Long X.D., Wang W., Jiao H.K., Mei Z., Yin Q.Q., Ma L.N., Zhou A.W., Wang L.S. (2014). Cbx4 governs HIF-1alpha to potentiate angiogenesis of hepatocellular carcinoma by its SUMO E3 ligase activity. Cancer Cell.

[84] Cashman R., Cohen H., Ben-Hamo R., Zilberberg A., Efroni S. (2014). SENP5 mediates breast cancer invasion via a TGFbetaRI SUMOylation cascade. Oncotarget.

[85] Bernstock J.D., Ye D.G., Lee Y.J., Gessler F., Friedman G.K., Zheng W., Hallenbeck J.M. (2018). Drugging SUMOylation for neuroprotection and oncotherapy. Neural Regen. Res..

[86] Mao H., Lebrun D.G., Yang J., Zhu V.F., Li M. (2012). Deregulated signaling pathways in glioblastoma multiforme: Molecular mechanisms and therapeutic targets. Cancer Investig..

[87] Bernstock J.D., Ye D., Gessler F.A., Lee Y.J., Peruzzotti-Jametti L., Baumgarten P., Johnson K.R., Maric D., Yang W., Kogel D. (2017). Topotecan is a potent inhibitor of SUMOylation in glioblastoma multiforme and alters both cellular replication and metabolic programming. Sci. Rep..

[88] Soares I.N., Caetano F.A., Pinder J., Rodrigues B.R., Beraldo F.H., Ostapchenko V.G., Durette C., Pereira G.S., Lopes M.H., Queiroz-Hazarbassanov N. (2013). Regulation of stress-inducible phosphoprotein 1 nuclear retention by protein inhibitor of activated STAT PIAS1. Mol. Cell Proteom..

[89] Wang L., Banerjee S. (2004). Differential PIAS3 expression in human malignancy. Oncol. Rep..

[90] Xia W., Tian H., Cai X., Kong H., Fu W., Xing W., Wang Y., Zou M., Hu Y., Xu D. (2016). Inhibition of SUMO-specific protease 1 induces apoptosis of astroglioma cells by regulating NF-kappaB/Akt pathways. Gene.

[91] Nakayama K.I., Nakayama K. (2006). Ubiquitin ligases: Cell-cycle control and cancer. Nat. Rev. Cancer.

[92] Tang S., Huang G., Tong X., Xu L., Cai R., Li J., Zhou X., Song S., Huang C., Cheng J. (2013). Role of SUMO-specific protease 2 in reprogramming cellular glucose metabolism. PLoS ONE.

[93] Agbor T.A., Cheong A., Comerford K.M., Scholz C.C., Bruning U., Clarke A., Cummins E.P., Cagney G., Taylor C.T. (2011). Small ubiquitin-related modifier (SUMO)-1 promotes glycolysis in hypoxia. J. Biol. Chem..

[94] Agnihotri S., Zadeh G. (2016). Metabolic reprogramming in glioblastoma: The influence of cancer metabolism on epigenetics and unanswered questions. Neuro. Oncol..

[95] Xu H., Rahimpour S., Nesvick C.L., Zhang X., Ma J., Zhang M., Zhang G., Wang L., Yang C., Hong C.S. (2015). Activation of hypoxia signaling induces phenotypic transformation of glioma cells: Implications for bevacizumab antiangiogenic therapy. Oncotarget.

[96] Iwadate Y. (2016). Epithelial-mesenchymal transition in glioblastoma progression. Oncol. Lett..

[97] Minata M., Audia A., Shi J., Lu S., Bernstock J., Pavlyukov M.S., Das A., Kim S.H., Shin Y.J., Lee Y. (2019). Phenotypic plasticity of invasive edge glioma stem-like cells in response to ionizing radiation. Cell Rep..

[98] Yang Y., Xia Z., Wang X., Zhao X., Sheng Z., Ye Y., He G., Zhou L., Zhu H., Xu N. (2018). Small-molecule inhibitors targeting protein sumoylation as novel anticancer compounds. Mol. Pharmcol..

[99] Huang W., He T., Chai C., Yang Y., Zheng Y., Zhou P., Qiao X., Zhang B., Liu Z., Wang J.J. (2012). Triptolide inhibits the proliferation of prostate cancer cells and down-regulates SUMO-specific protease 1 expression. PLoS ONE.

[100] Uno M., Koma Y., Ban H.S., Nakamura H.J.B. (2012). Discovery of 1-[4-(N-benzylamino) phenyl]-3-phenylurea derivatives as non-peptidic selective SUMO-sentrin specific protease (SENP) 1 inhibitors. Bioorg. Med. Chem. Lett..

[101] Xie W., Wang Z., Zhang J., Wang L., Zhao Y., Zhou H. (2016). Development and evaluation of a highly reliable assay for SUMO-specific protease inhibitors. Bioorg. Med. Chem. Lett..

[102] Chen Y., Wen D., Huang Z., Huang M., Luo Y., Liu B., Lu H., Wu Y., Peng Y., Zhang J. (2012). 2-(4-Chlorophenyl)-2-oxoethyl 4-benzamidobenzoate derivatives, a novel class of SENP1 inhibitors: Virtual screening, synthesis and biological evaluation. Bioorg. Med. Chem. Lett..

[103] Brave M., Dagher R., Farrell A., Abraham S., Ramchandani R., Gobburu J., Booth B., Jiang X., Sridhara R., Justice R. (2006). Topotecan in combination with cisplatin for the treatment of stage IVB, recurrent, or persistent cervical cancer. J. Oncol..

[104] Pommier Y. (2006). Topoisomerase I inhibitors: Camptothecins and beyond. J. Nat. Rev. Cancer.

[105] Ling Y.-H., Donato N.J., Perez-Soler R. (2001). Sensitivity to topoisomerase I inhibitors and cisplatin is associated with epidermal growth factor receptor expression in human cervical squamous carcinoma ME180 sublines. J. Cancer Chemother. Pharmacol..

[106] Mo Y.-Y., Yu Y., Shen Z., Beck W.T. (2002). Nucleolar delocalization of human topoisomerase I in response to topotecan correlates with sumoylation of the protein. J. Biol. Chem..

[107] Rapisarda A., Uranchimeg B., Scudiero D.A., Selby M., Sausville E.A., Shoemaker R.H., Melillo G. (2002). Identification of small molecule inhibitors of hypoxia-inducible factor 1 transcriptional activation pathway. J. Cancer Res..

[108] Hirohama M., Kumar A., Fukuda I., Matsuoka S., Igarashi Y., Saitoh H., Takagi M., Shin-ya K., Honda K., Kondoh Y. (2013). Spectomycin B1 as a novel SUMOylation inhibitor that directly binds to SUMO E2. ACS Chem. Biol..

[109] Kho C., Lee A., Jeong D., Oh J.G., Gorski P.A., Fish K., Sanchez R., DeVita R.J., Christensen G., Dahl R. (2015). Small-molecule activation of SERCA2a SUMOylation for the treatment of heart failure. Nat. Commun..

[110] Bernstock J.D., Lee Y.-j., Peruzzotti-Jametti L., Southall N., Johnson K.R., Maric D., Volpe G., Kouznetsova J., Zheng W., Pluchino S. (2015). A novel quantitative high-throughput screen identifies drugs that both activate SUMO conjugation via the inhibition of microRNAs 182 and 183 and facilitate neuroprotection in a model of oxygen and glucose deprivation. J. Cereb. Blood Flow Metab..

[111] Schneekloth J.S. (2017). Controlling protein SUMOylation. Nat. Chem. Biol..

[112] He X., Riceberg J., Soucy T., Koenig E., Minissale J., Gallery M., Bernard H., Yang X., Liao H., Rabino C. (2017). Probing the roles of SUMOylation in cancer cell biology by using a selective SAE inhibitor. Nat. Chem. Biol..

[113] Fukuda I., Ito A., Hirai G., Nishimura S., Kawasaki H., Saitoh H., Kimura K., Sodeoka M., Yoshida M. (2009). Ginkgolic acid inhibits protein SUMOylation by blocking formation of the E1-SUMO intermediate. Chem. Biol..

[114] Fukuda I., Ito A., Uramoto M., Saitoh H., Kawasaki H., Osada H., Yoshida M. (2009). Kerriamycin B inhibits protein SUMOylation. J. Antibiot..

[115] Takemoto M., Kawamura Y., Hirohama M., Yamaguchi Y., Handa H., Saitoh H., Nakao Y., Kawada M., Khalid K., Koshino H. (2014). Inhibition of protein SUMOylation by davidiin, an ellagitannin from Davidia involucrata. J. Antibiot..

[116] Suzawa M., Miranda D.A., Ramos K.A., Ang K.K., Faivre E.J., Wilson C.G., Caboni L., Arkin M.R., Kim Y.S., Fletterick R.J. (2015). A gene-expression screen identifies a non-toxic sumoylation inhibitor that mimics SUMO-less human LRH-1 in liver. eLife.

[117] Wu J., Lei H., Zhang J., Chen X., Tang C., Wang W., Xu H., Xiao W., Gu W., Wu Y. (2016). Momordin Ic, a new natural SENP1 inhibitor, inhibits prostate cancer cell proliferation. Oncotarget.

[118] Schulman B.A., Wade Harper J. (2009). Ubiquitin-like protein activation by E1 enzymes: The apex for downstream signalling pathways. Nat. Rev. Mol. Cell Biol..

[119] Zhao B., Villhauer E.B., Bhuripanyo K., Kiyokawa H., Schindelin H., Yin J. (2014). SUMO-mimicking peptides inhibiting protein SUMOylation. ChemBioChem Eur. J. Chem. Biol..

